# Valence and Intensity of Video Stimuli of Dogs and Conspecifics in Sheep: Approach-Avoidance, Operant Response, and Attention

**DOI:** 10.3390/ani8070121

**Published:** 2018-07-17

**Authors:** Camille M. C. Raoult, Lorenz Gygax

**Affiliations:** 1 Centre for Proper Housing of Ruminants and Pigs, Federal Food Safety and Veterinary Office FSVO, Agroscope, Tänikon 1, 8356 Ettenhausen, Switzerland; camille.raoult@agroscope.admin.ch; 2 Animal Welfare Division, Veterinary Public Health Institute, Vetsuisse Faculty, University of Bern, Länggassstrasse 120, CH-3012 Bern, Switzerland; 3 Animal Husbandry, Albrecht Daniel Thaer-Institute of Agricultural and Horticultural Sciences, Humboldt-Universität zu Berlin, Unter den Linden 6, 10115 Berlin, Germany

**Keywords:** sheep, valence, video stimuli, approach–avoidance paradigm, operant conditioning, attention

## Abstract

**Simple Summary:**

Animals’ judgement of stimuli’s negativity or positivity cannot always be assumed. To assess the valence and intensity of video stimuli (dogs as negative vs. conspecifics as positive stimuli) in sheep, we used three experimental approaches: (1) an approach-avoidance paradigm; (2) operant conditioning using the videos as reinforcers; and (3) an attention test. We measured sheep’s behavioral and physiological reactions. Sheep generally reacted to the videos presented. Nevertheless, we found no support with the approach–avoidance paradigm, and the attention test for dog videos were more negative than sheep videos. However, the operant conditioning indicated that sheep were more prone to avoid videos of moving dogs. Overall, we found that standard video images may not be ideal to represent valence characteristics of stimuli to sheep.

**Abstract:**

Stimuli are often presumed to be either negative or positive. However, animals’ judgement of their negativity or positivity cannot generally be assumed. A possibility to assess emotional states in animals elicited by stimuli is to investigate animal preferences and their motivation to gain access to these stimuli. This study’s aim was to assess the valence of social stimuli in sheep. We used silent videos of varying intensity of dogs as negative versus conspecifics as positive stimuli in three approaches: (1) an approach–avoidance paradigm; (2) operant conditioning using the video stimuli as reinforcers; and (3) an attention test. In the latter, we assessed differential attention of sheep to simultaneous projections by automatically tracking sheep head and ear postures and recording brain activity. With these approaches, it was difficult to support that the sheep’s reactions varied according to the stimuli’s presumed valence and intensity. The approach–avoidance paradigm and attention test did not support the assumption that dog videos were more negative than sheep videos, though sheep reacted to the stimuli presented. Results from the operant conditioning indicated that sheep were more prone to avoid videos of moving dogs. Overall, we found that standard video images may not be ideal to represent valence characteristics of stimuli to sheep.

## 1. Introduction

In animal welfare research, the interest increasingly focuses on affective states [1], and more particularly, on positive emotions [2,3]. Emotions are often triggered by external stimuli [4,5]. These stimuli can vary in valence (negative vs. positive) and arousal (calming vs. exciting). These characteristics are either considered to be independent [6,7,8] or arousal is seen as an integral component of valence, that is, the intensity of the stimulus [9,10]. There seem to be indicator variables that reflect arousal quite well, but it is notoriously difficult to find indicators of valence. Moreover, situations of opposite valence but similar arousal are difficult to come up with [11], though they would be of great experimental value. Different stimuli are presumed to be either negative, such as frustration (unpalatable food reward [8,12,13]), isolation (separation from group members [14,15,16]), or predator presentation [17,18,19], or positive, such as feed reward [14,20,21] or grooming [13,22,23,24]. However, the negativity or the positivity of the stimuli are not always clear; the animals’ judgement of these stimuli cannot be assumed easily a priori [24].

Contrary to humans, which are able to verbally express their feelings, other approaches are needed in animals. Both behavioral and physiological (e.g., heart rate or brain activity) indicator variables have been proposed [2,25,26,27,28]. Alternatively, animal preferences and their motivation to gain access to stimuli can be investigated in experimental tests [29,30]. Moreover, negative and positive stimuli can be used as punishers or reinforcers, respectively, in operant conditioning paradigms [31]. Finally, the bias in attention that an animal shows towards a simultaneously presented pair of positive and negative stimuli may reflect the relative strength of these two stimuli [28,32,33,34,35].

The valence of a stimulus is reflected by the motivation to avoid or approach an aversive or appetitive stimulus, respectively [29,36]. The approach–avoidance paradigm is often used to estimate the strength of aversion, by presenting a neutral or “aversive” stimulus (e.g., human, dog) between the tested animal and an “attractive” stimulus (e.g., group of conspecifics, food reward). Placing the two stimuli next to each other generates conflicting motivations and the tested animal places itself at a distance that is a compromise between being too close to the aversive stimulus and too far away from the attractive stimulus [37]. The approach–avoidance test has been used in sheep to measure aversion to humans [17,18,37,38,39], a real dog [18], a real goat [18], and a simple box [18], but also averseness of a handling procedure [40].

Operant conditioning is a learning process in which behavior is modified through the use of positive and negative reinforcement [41]. In an operant task, an animal learns to perform a particular behavior to access a specific resource or avoid an unwanted outcome [31]. According to McLeod [41], behavior that is positively reinforced tends to be repeated, while behavior that is not reinforced tends to be extinguished. Operant studies have been conducted in sheep on behavioral thermoregulation, illumination preference and odors discrimination [31,42,43], as well as to investigate sodium appetite (for a review see Reference [31]).

The attention that an animal shows towards a stimulus can be used in an experimental test as an indicator of the valence of the stimulus. Attention bias tests have been used to show increased allocation of attention towards threat-related stimuli in anxious compared with non-anxious individuals. Attention can be assessed by the time an animal looks at a given stimulus. It is assumed that animals gaze towards objects or scenes depending on their relevance [44]. Moreover, attention can be assessed by kinematic indicators [45], such as the movements and specific positions of the ears and head. Several studies in sheep [10,13,14,28,46,47,48,49,50,51,52,53,54] have recorded head and ear postures and position changes as measures of affective state, which are likely to be related to attention.

Different kinds of stimuli could be used to assess attention, and in sheep, visual stimuli are thought to be relevant. Presenting videos instead of real animals as stimuli allows the experimenter to standardize the stimuli presented by manipulating the content and intensity of the scenes [55,56]. In previous studies using visual stimuli, sheep were shown to be able to discriminate faces of different sheep, sheep breeds, humans, dogs, and goats using back-projected images [18,57,58,59,60]. Moreover, it was found that two-dimensional life-size images are salient stimuli for sheep, and that stimuli work best if subjects are shown in frontal view [38,58,61]. Sheep particularly use their binocular vision to accurately see when they detect movement [62,63], and their vision was found to be better for moving rather than static objects [64,65,66], possibly because they are prey animals and have to be attentive to movements of predators [67]. To inspect a stimulus closely, sheep may orient their head to perceive it with their binocular field of vision (around 40–60° where both eyes can see, References [62,68] cited and confirmed by Piggins and Phillips, 1996). Given the wide visual angle of sheep (monocular vision fields around 313° [62,63]), however, they may not always orient their head towards a stimulus to focus on it. Therefore, additional outcome variables, such as ear movements and postures, as well as cortical activation, might be valuable to measure the attention sheep pay to a given stimulus.

Previous works studied the valence of stimuli in sheep. Presenting images of sheep faces has been shown to reduce stress in sheep [38], indicating that sheep can be considered as a positive stimulus. Vögeli, Wolf, Wechsler, and Gygax [48] also reported differential reactions of sheep in response to video sequences of different types of social interactions by conspecifics, with sheep’s attentiveness decreasing monotonously from video sequences showing agonistic interactions to ruminating sheep to co-feeding sheep. Additionally, it is commonly assumed that humans and dogs are species that would normally represent some form of threat for sheep [28,46,58,69,70].

In this study, we investigated whether and how sheep assess video stimuli with respect to their valence. We used stimuli reflecting other animals (inter- and intraspecific) and used predators (dogs) as a presumed negative stimulus versus conspecifics as a presumed positive stimulus. We hypothesized that sheep could distinguish between sheep and dogs and their specific behaviors on video images. We implemented several experimental approaches. First, an approach–avoidance paradigm was used. We hypothesized that sheep would be slower to leave the start box and to cross the screens showing the dog videos when the video intensity increased. Second, we used video stimuli as reinforcers in an operant conditioning situation. We hypothesized that sheep would be motivated to work for watching sheep videos and would try to avoid dog videos. Third, we developed an attention test in which two videos showing dogs or sheep were presented simultaneously. We hypothesized that sheep would pay attention to both stimuli, but be especially interested in videos showing dogs or sheep at close distance and videos presenting more intensive stimuli.

## 2. Materials and Methods

### 2.1. Animals

Thirty–three non-lactating and non-reproducing Lacaune female sheep (born between January and March 2016) were housed in eight group pens (2.4 m × 3.5 m per group of four sheep, with one group of five) in an open-front barn at the Agroscope Research Station in Tänikon, Switzerland. They had straw bedding available, hay was provided twice a day at a regular time, and water was available ad libitum. The sheep had previous visual contact with the farmer’s herding dogs, at an age younger than 5 months. At the start of the testing period, sheep were from 9 to 12 months old.

All the animals were habituated to being handled by an experimenter (CR). Each group of four sheep was then familiarized with a mobile pen (2.3 m × 2 m wooden structure without floor and mounted on wheels), used to move the group from the home pen to the test arenas.

In each experiment, tests for a given sheep (or pair of sheep) took place at the same time of day to control for any potential daily periodicity in the sheep’s reactions.

This study was approved by the Research Commission of the Federal Food Safety and Veterinary Office, and the necessary authorization to conduct animal experiments was granted by the cantonal authorities (Canton of Thurgau permit no. 27508-TG01/16).

### 2.2. General Statistical Aspects

Statistical analyses were performed in R version 3.4.2. (for experiments 1 and 2 [71]) and version 3.3.1. (for experiment 3 [72]) using linear mixed-effects models (lmer) for continuous outcome variables and generalized linear mixed-effects models (glmer, package lme4 [73]) for dichotomous outcome variables. Statistical assumptions were checked using a graphical analysis of residuals focusing on the distribution of errors and random effects, and homoscedasticity of errors of the models (package DHARMa [74]). For continuous outcome variables, *p*-values were calculated using parametric bootstraps (package pbkrtest [75]). In contrast, we used the likelihood-ratio test to calculate *p*-values for the dichotomous outcome variables (package lme4 [73]) because the bootstraps did not converge well enough. With the first two experimental approaches, we followed a full model approach [76]. For this, we first calculated the global *p*-value (between the maximum and the null model). If that model reached a low *p*-value, we tested each of the predictor variables singly by comparing the full model to the one omitting this predictor. In order to allow for meaningful comparisons for main effects when interactions formed part of the model, we used sum-contrasts for our predictor variables. Model predictions were calculated based on semi-parametric bootstraps for mixed models (package boot [77]).

### 2.3. Experiment 1: Appraoch-Avoidance Paradigm

#### 2.3.1. Animals

In this experiment, 24 sheep were tested. Six of the eight groups of sheep were randomly chosen to reach a sample size that had provided sufficient power for a relevant effect size in previous studies [22,47,78]. The experiments took place between November 2016 and January 2017. Before that, and to develop the experimental procedure described, a series of pilot trials was conducted using the eight sheep of the two remaining groups.

#### 2.3.2. Habituation, Experimental Procedure and Video Stimuli

**Figure 1:**
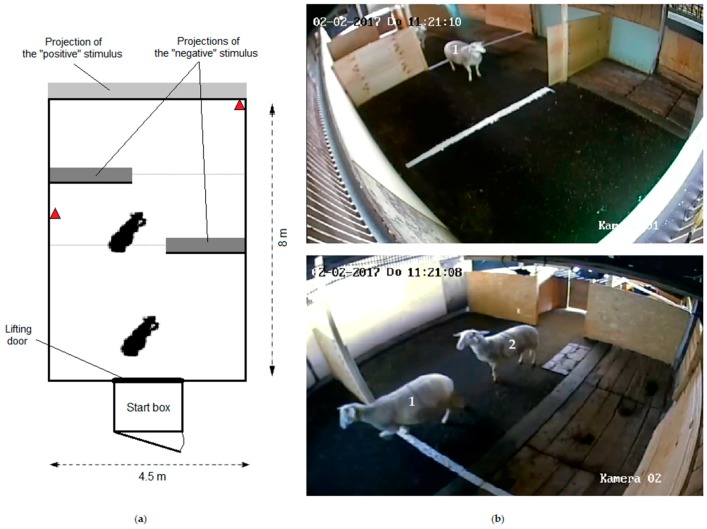
Test arena for the approach–avoidance paradigm: (**a**) Plan of the test arena and position of the two cameras indicated by red triangles; (**b**) Pictures of the test arena captured by the two cameras during a trial, with sheep 1 moving between the two “negative” screens and sheep 2 between the start box and the first negative screen.

The sheep were habituated in pairs once a day during 4 days to the indoor test arena (36 m^2^; Figure 1) and its start box (1.20 m^2^, with a lifting door; Figure 1). The time in the pen increased from 90 s on the first to 300 s on the fourth day.

Sheep were tested in pairs also because they were found to be too nervous if alone in the pilot trials. Pairs were defined during the first trial in the habituation phase and remained constant over the test trials. On each day, the pair was separated and moved into the start box of the test arena, where sheep were allowed to calm down for 30 s. Then they were given access to the test pen for 90 s per trial. After that they returned to the start box from which they were released to the next trial after approximately 45 s. The four trials of a day took place one immediately after the other.

A presumed positive stimulus was projected on the back wall of the test arena in order to elicit an approach reaction, while a presumed negative stimulus was projected on each of two smaller screens that formed a very simple type of maze (Figure 1). The video on the two smaller screens was identical in any given trial. To provoke and assess the reaction in the sheep, stimuli differing in their presumed valence and intensity were presented as silent video images (see Reference [48]). For the presumed positive stimuli, the videos were of unfamiliar sheep, i.e., unknown sheep of the same breed, age, and sex. Videos showing one (low intensity) or several (high intensity) sheep grazing, walking, or engaged in affiliative social interactions (licking or rubbing against each other) were used. With presumed increasing intensity, videos showing an unknown dog lying calmly, sitting with the face directed towards the sheep, moving (filmed from the side while the dog was following or coming in the direction of a human experimenter) and eventually barking, or two dogs moving and eventually barking were used for the presumed negative valence. For a pair of sheep, the same dog was shown in all videos of a given day. Different breeds of dog were used in the videos: a Beauce dog (in pair with another Beauce dog), a White Swiss Shepherd (in pair with a Dalmatian), and a Flat Coated Retriever crossbreed (in pair with a Podengo crossbreed). Each tested pair of sheep was submitted to at least two breeds of dog in the videos of their 4 days of experiments. During the tests, the same video was only played once per pair of sheep to avoid any habituation to the video presentation. Videos had been taken with a digital video camera recorder (Sony DCR-SX33E, resolution 720 × 576 pixels, Sony Corporation, Tokyo, Japan) and were repeated in direct succession to reach the necessary length of 90 s. Pieces of videos lasting between 2 to 90 s (18 s on average) were used. Videos were projected with projectors (a Hitachi CP-RX70, Hitachi Construction Machinery Co. Ltd., Tokyo, Japan, at 25 fps at 1024 × 768 pixels resolution using Liquid Cristal Display (LCD) technology, and a Dataview S240, Visinfo AG, Baden-Dättwil, Switzerland, at 25 fps at 1024 × 768 pixels resolution using Digital Light Processing (DLP) technology, for the dog video projections, and an Epson multimedia Projector EB-1761W, Seiko Epson Corporation, Nagano, Japan, at 25 fps at 1024 × 768 pixels resolution using LCD technology, for the sheep video projections) onto the screens that consisted of wooden boards that were painted white. Projected animals in the videos were about natural size.

Each pair of sheep was tested on 4 consecutive days and was confronted on each day to four video sequences with the same presumed intensity regarding the conspecifics and the four presumed intensity levels regarding the dogs. Accordingly, each pair was subjected to 16 different video sequences in total. Each combination of sheep and dog video intensity was presented twice to each pair, and the order of the sequences was balanced across the pairs of sheep.

#### 2.3.3. Behavioral Measurements

During the presentation of video sequences, the behavior of the sheep was recorded for each individual in a pair, using direct observation and video recordings. Indicator variables for locomotor activity and attention were recorded. To define some of the reactions, the concrete floor of the test arena was divided into three sections in relation to the position of the three screens (Figure 1). The latency to leave the start box (i.e., both front legs outside), the latency to cross the first line aligned with the first projection screen, the latency to cross the second line, and the latency to re-cross the second line (no front legs beyond this line) was measured using a stop watch. Additionally, the duration (in seconds) of attention towards both the negative screens and the positive screen was observed. This was scored when the head of a sheep was up with the ears forward and a view angle matching to the screen watched, that is, the nose pointed towards the screen. Also, specific behavioral reactions of the sheep directed at the screens were noted, that is, whether the sheep backed away from the video projections, smelled the screens, or showed sign of stress (urination or defecation). Intra-observer agreement was assessed in 48 trials. To do so, two trials per pair of sheep were randomly chosen and assessed a second time one week later for both sheep by the same observer.

#### 2.3.4. Statistical Analysis

We evaluated the proportion of the latency to leave the start box, the proportion of time spent paying attention towards the negative screens, and the proportion of time spent paying attention towards the positive screen in relation to total trial duration as continuous outcome variables. All these proportions were logit transformed for the evaluation. Whether the first or second line was crossed and whether the second line was re-crossed were used as dichotomous outcome variables. We did not evaluate the latencies to cross these lines as continuous variables because a high number of individuals did not cross them and a meaningful proportion of the data would then have been censored. Proportions of time spent smelling the negative screens and smelling the positive screen were also dichotomized due to their rare occurrence. Defecation or urination occurred too rarely for a quantitative analysis. Additionally, the pattern for sheep crossing the second line and re-crossing the second line were very similar because most of the sheep (98.45%) re-crossed the second line after they went to the back-screen. Therefore, we show the results for the sheep that crossed the second line only.

The maximum model included the presumed intensity of the negative stimulus (factor with four levels), the presumed intensity of the positive stimulus (factor with two levels), and their interaction as fixed effects. For the continuous outcome variables, the random effects were composed of four crossed parts: (1) the sheep identity was nested in pair and nested in housing group to account for the repeated measurements of the sheep, and (2) the trial ID nested within test day was included to reflect the fact that the sheep in a pair were observed at the same time and several pairs were tested on the same day. In addition, (3) the identity of the sheep video nested in the sheep video intensity, and (4) the identity of the dog video nested in the dog video intensity nested in the dog identity, were included to account for the variability between the different videos used. For the dichotomous outcome variables, the random effects consisted of the sheep identity only. The models with dichotomous outcomes were kept simpler in this respect because these models were over-specified as seen with problems of convergence in estimating the models if they included a more complex random effects structure. This is likely to be anti-conservative to some extent because some of the dependencies in the data were not considered then.

To check intra-observer agreement we used Cohen’s kappa on the variables: whether the first line was crossed, whether the second line was crossed, whether the negative screens were smelled, and whether the positive screen was smelled (package psych [79]). We used “agreement” on the variables: latency to leave the start box, time spent paying attention towards the negative screens, and attention towards the positive screen (package Agreement [80]).

### 2.4. Experiment 2: Operant Conditioning

#### 2.4.1. Animals

In this experiment, 16 sheep were tested singly. Four of the eight groups of sheep available were randomly chosen. The tests took place in March 2017.

#### 2.4.2. Experimental Procedure

In preparation for the operant conditioning approach, sheep had been trained regularly over nine months to touch a target, i.e., a wooden stick with a yellow tennis ball at its end, to obtain food. This target was also used when we wanted the sheep to follow the experimenter.

In the experiment, sheep could switch a presumed positive video (i.e., sheep video) on for 5 s or a presumed negative video (i.e., dog video) off for 5 s when they touched the target with their muzzle. Sheep did not obtain a food reward anymore when touching the target. Each time the sheep touched the target, it was moved out of sight for 5 s. The videos shown were unknown to the tested sheep but were the same as the ones used in the first experiment. We used only two levels of intensity for the dog videos (a single dog lying calmly, and a single dog moving and eventually barking). Each video was shown only during one specific trial for each sheep. They were projected (Epson multimedia Projector EB-1761W, Seiko Epson Corporation, Nagano, Japan) onto a screen that was painted white and presented such that the animals in the video were about natural size. Each video stimulus was continuously repeated as in experiment 1 for a trial duration of 180 s. Pieces of videos lasting between 2 to 97 s (23 s on average) were used.

Experimental testing was performed in an indoor test pen (2.5 m × 1.5 m) with the screen on the narrow wall. This pen was situated close to the sheep barn without visual contact with the other sheep. The sheep were tested singly, with a human experimenter in view, and could move freely inside the test pen. Each sheep was subjected to one daily trial for 4 consecutive days per week. In total, they were shown four different video stimuli, with only one stimulus tested each week (and four repetitions). Balance across sheep was ensured: they started with either two weeks of sheep or dog videos. Within these two weeks, the sequence of low and high intensity was again balanced across sheep. Because sheep did not pay attention to the videos consistently in the first week of testing, we decided to consider this week as a training week, and we ran the first experimental condition across a second week. Moreover, we included an additional test week at the end, where the sheep’s action had a reverse effect on the video, i.e., the sheep switched on the dog video or switched off the sheep video when touching the target. This happened for the same videos they had seen in week 5.

#### 2.4.3. Statistical Analysis

The number of target touches was log-transformed and analyzed as a continuous outcome variable. We analyzed our data in three distinct parts (using subsets): week 1 (i.e., habituation phase), weeks 2 to 5 (i.e., experimental changes), and weeks 5 and 6 (i.e., reversal phase). For the reversal phase, we decided to analyze dog and sheep videos in separate models since the sheep’s responses looked very different graphically and the interaction between the effect of target touch and the valence reached a *p*-value of 0.06 indicating a potential interaction (see Results 3.2).

For week 1 and weeks 2–5, the maximum model included the day of test within the week (factor with four levels), presumed intensity (factor with two levels) and valence of the stimuli (factor with two levels), and their interactions, as fixed effects. For weeks 5–6, the maximum model included the day of test within the week (factor with four levels), presumed intensity (factor with two levels) and valence of the stimuli (factor with two levels), effect of target touch (i.e., normal or reversed; factor with two levels), and their interactions, as fixed effects. For the three parts, the random effects included the sheep identity nested in the housing group to account for the repeated measurements of the sheep, and the video identity nested in the video intensity nested in the video valence to account for the different videos.

### 2.5. Experiment 3: Attention Test

#### 2.5.1. Animals

In this experiment, we used 32 sheep. Pilot trials were conducted on four sheep (from one randomly chosen group of the eight available groups) in order to verify that sheep can be familiarized (i.e., in less than two weeks) to wear a hood and dummy measurement devices and be habituated to be restrained (see Section 2.5.2). This group was not used in the formal experiment, so only 28 sheep were tested. Sheep were habituated either 0 (*n* = 9, unhabituated sheep), 5 (*n* = 10), or 9 (*n* =9) times in order to find out for future practical application whether and how much sheep needed to be habituated to perform the test, and whether the same results would be found without any habituation at all. The experiments took place in September and October 2017.

#### 2.5.2. Habituation and Experimental Procedure

**Figure 2:**
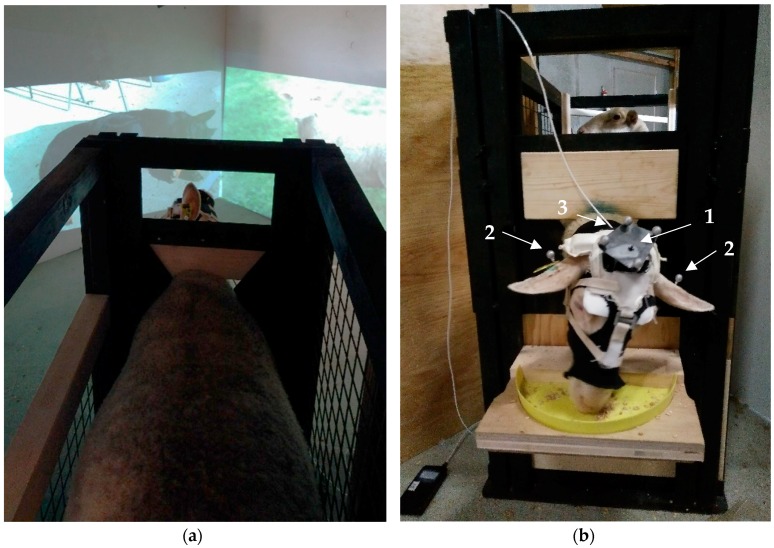
Test arena for the attention test: (**a**) the sheep was restrained in a feeding station; and (**b**) equipped with the different layers of the hood and the measurement devices: (1) head target; (2) ear targets; (3) fNIRS sensor below the hood.

Nine sheep were unhabituated before testing while the 19 other sheep were familiarized to being restrained in a box and wearing a hood in their home pen for 10 min a day on 5 consecutive days. The hood consisted of: (1) a legging cut length-wise to cover the head and neck of the sheep, with four holes for the eyes and ears and a small window to keep the functional near infrared spectroscopy (fNIRS) sensor fixated on the sheep scalp, (2) a black plastic sheet sewn in tissue to avoid any interference between natural light and the fNIRS sensor, (3) a football gaiter tightened by four Velcro fasteners put on to stabilize and protect the previous layers on the head of the sheep, and (4) a halter of cloth with a metal bridge screwed on it to cover the fNIRS sensor, as well as to attach the head target (see Figure 2b). The hood was pulled over the head of the animal for a tight and secure attachment of the devices to be used (Figure 2b; adapted from Reference [48]).

In the following two weeks, 10 and 9 sheep were individually habituated to the testing environment and the measurement devices for 10 min once a day (either in the morning or in the afternoon) on 5 and 9 days, respectively.

The testing environment (Figure 2) consisted of a feeding station, i.e., a box of the size of a single animal to restrict the sheep’s movement (1 m × 0.6 m) with closed walls on the side and a trough at its narrow end. The trough was filled with food before the session to induce feeding in the animals such that they kept their head towards the area where stimuli were presented, and were focused on the feed, at least at the start of each session (e.g., some sheep had eaten the food 30 s after the beginning, while others still had food at the end of the session). The trough was open to a space in which two projection screens were visible at a right angle. This test arena was situated close to the sheep barn without visual contact of the other sheep.

The sheep were tested in four batches in a total of three consecutive weeks. The experiment of the first batch of six sheep started on the Monday of the week following the habituation weeks. The unhabituated sheep were tested 2 days after the last habituated sheep were tested, in a fourth batch of nine sheep. Within a batch, each sheep underwent one daily session on 4 consecutive days. The interval between the habituation and the experiment varied with batch. However, this was balanced by testing sheep that were habituated 5 and 9 days in each batch.

On each day, a complete housing group of four sheep was brought to the test arena and they left the mobile pen to enter a waiting zone (2.6 m × 1.5 m) voluntarily. The first sheep to be tested entered the test apparatus box voluntarily, or was picked out singly and guided into the box. There, it was equipped with the measurement devices and left quietly for 60 s of acclimatization during which it could eat (a mixture of UFA 763 ProRumin COMBI QM, Herzogenbuchsee, Switzerland). Then, the sheep went through a test session with two blocks of nine trials each. The presumed negative and positive stimuli were simultaneously projected, each on one of the two screens, for 30 s per trial. The two blocks within a session varied with respect to the side of the stimuli, i.e., half of the sheep started with the presumed negative stimulus on the left and the presumed positive stimulus on the right side. The side of the stimuli was balanced across sheep and sessions. The nine trials of a block took place one immediately after the other and consisted of all possible combinations of presenting the two stimuli at different distances and intensities. Animal(s) on the video were either in foreground, middle ground, or background. That is, the trials varied the absolute distance to the stimuli and the relative distance between the stimuli. The nine trials were presented in random order using another sequence each time. The inter-trial intervals varied randomly between 15 and 21 s to avoid temporal anticipation. During these intervals a black screen was presented. Across the four sessions, the intensity of both stimuli varied in a 2 × 2 crossed design, i.e., both stimuli were presented in two intensities (low and high) by varying the number and behavior of the animals in the stimulus sequences. Each sheep was subjected to 18 different video sequences a day over 4 days (a total of 72 sequences).

For this experiment, new videos were taken with the same digital video camera recorder used before (Sony DCR-SX33E, resolution 720 × 576 pixels, Sony Corporation, Tokyo, Japan) and displayed as silent video images. The same type of sheep and dog videos were used as in the first two experiments but at different distances, i.e., animal(s) on the videos were shown in about natural size for being in the foreground, about 25% of natural size for being in the middle ground, or about 1/8 of natural size for being in the background. The same sheep video intensities (i.e., one or several unknown sheep) and dog video intensities (i.e., one dog lying calmly and one dog moving and eventually barking) were selected as in the experiment using the operant conditioning approach. The dog videos showed different breeds of dogs: a Beauce dog (same as before), a White Swiss Shepherd (same as before), a Cavalier King Charles-Pointer crossbreed, and a Dalmatian. Each tested sheep was subjected to videos of all four dogs over the trials. A given video was shown to the sheep only during one specific trial to avoid any habituation to the video presentation. They were projected (two Epson multimedia Projector EB-1761W, Seiko Epson Corporation, Nagano, Japan, for the right and left side) onto wooden walls that were painted white. Each video stimulus was repeated in direct succession to reach the necessary length of 30 s. Pieces of videos lasting between 1 to 22 s (10 s on average) were used.

The head of the habituated sheep were sheared on the eve of the first day of the experiment to enhance the skin contact of the fNIRS sensor using a hand-shearing machine. As described by Vögeli, et al. [81], the sheep heads were then depilated using a human depilation cream (Veet Aloe Vera depilation cream, Reckitt Benckiser Health care (UK) Ltd., Hull, UK) to avoid any interference between the hair and the sensor (light-piping). According to Vögeli, Wolf, Wechsler, and Gygax [81], epilation could lead to inflammation. We did not observe such inflammations, and even if a rare subclinical inflammation occurred, this would have been part of the inter-individual or day-to-day variability accounted for in the model.

#### 2.5.3. Behavioral Measurements: Head Orientation, Ear Movements and Postures

Attention was assessed by measuring head orientation to one of the screens, as well as ear movements and postures [47,48,81,82]. These variables were recorded during the test by an automated tracking system (TRACKPACK/E, Advanced Realtime Tracking ART System, Weilheim, Germany). The system consisted of four infrared-sensitive cameras (3.5 mm focal lens, IR flash 850 nm) fitted above the test apparatus, a head target composed of four reflective marker balls (Ø16 mm, 2.6 g each) in a fixed arrangement attached on top of the sheep’s head and centered between the ears (so-called 6-D), and two single ear targets composed each of one reflective marker ball fixed by a screw on the backside of the earmark (so-called 3-D; Figure 2b). The ear targets could be located in absolute 3-D space. Likewise, the specific configuration of the head target allowed both determining its absolute location in 3-D space and the sheep head orientation (roll, pitch, and yawn angles). The recorded sampling rate of 60 Hz was reduced to 6 Hz for analyses. The relative position of the ears was calculated in relation to the head (see also References [47,82]). For each trial composed of a 30 s stimulus and its respective pre- and post-stimulus phases (each lasting 6 s), we calculated the amount of head movements (sum of the absolute differences between successive horizontal angles divided by length of phase), as well as the relative number of head side changes (yawn angle switches from one side to the next), and the proportion of time that sheep were looking towards the right side (yawn angle more to the right than the direction straight ahead). We also calculated the amount of ear movements (sum of the absolute differences between successive horizontal angles of both ears divided by length of phase), the proportion of time when both ears pointed forwards (forward ears; both ears pointed more than 0 horizontal degrees forwards), the proportion of time when both ears were in a backwards position (backward ears; both ears pointed more than 10 horizontal degrees backwards), the proportion of time ears were relaxed (passive ears; vertical angle more than 30 degrees below the horizontal) and the proportion of time that the left ear was positioned more to the front than the right ear (left-asymmetric ears; left ear positioned at more than 5 degrees horizontal more forward than the right ear per all ear positions with more than 5 degrees difference in their horizontal angle) [82]. Potential indirect effects of the hood and targets on ear positions and movements was taken into account by the within-subject design of the experiment (see Statistical Analysis).

#### 2.5.4. Frontal Cortical Activation

The frontal cortical activity was recorded, except for the unhabituated sheep that were not equipped with the fNIRS sensor as it was difficult to do without habituation. Neural activity during stimulation is reflected by cortical oxygenation changes. The frontal cortical activation was measured by functional near-infrared spectroscopy (fNIRS) using an OxyPrem device (Biomedical Optics Research Laboratory, Zurich, Switzerland [83,84]). This device could measure haemodynamic changes in the brain, that is, the changes in the concentration of oxy- ([O_2_Hb]) and deoxyhaemoglobin ([HHb]) [22]. Brain activation is usually reflected by a concurrent increase in [O_2_Hb] and decrease in [HHb] [47,85]. The fNIRS sensor (9.0 cm × 4.0 cm) consisted of two photodetectors and four light sources emitting infrared light at three wavelengths each (LEDs at 760, 805, and 870 nm; source-detector distances of 15 and 25 mm), that is, eight light-paths in total which penetrated localized volumes of the cerebral cortex (for more details see Reference [22]). OxyPrem employs a self-calibrating principle using multiple light paths [86] for superior precision [87]. The recorded sampling rate of 100 Hz was reduced to 1 Hz for analyses. Just before the test, the sensor was positioned on the frontal part of the depilated sheep’s head and was kept in place by the hood.

#### 2.5.5. Statistical Analysis

Head and ear movements, ear postures, and changes in [O_2_Hb] and [HHb] as described above were used as outcome variables. Movements of the sheep’s head and ears, as well as the relative number of head side changes were log-transformed, and all the proportions (sheep looking towards the right side, both ears forward, and left-asymmetric ears) were logit-transformed for use as continuous outcome variables in one model for each outcome. [O_2_Hb] and [HHb] showed a distribution with long tails in the lower and upper value range that could not be assigned to either specific animals or video conditions. Therefore, according to Gygax, Reefmann, Wolf, and Langbein [12], we applied a transformation that shrinks both of these tails to the center of the distribution.

Because there were many more potential effects compared with the other experiments, we do not present a full model here. Instead we chose the statistical models for presentation based on their ranking according to the model weights derived from the Bayesian information criterion (BIC). The models selected by the BIC were simpler and likely to be more appropriate than those chosen by the more-classic Akaike information criterion (AIC) because we were interested in the causal relationship between predictors and outcome variables rather than in model predictions (for further details see References [12,88]). The model weights reflected the probability of each model being the best-fitting model within the given set of models (model probability, *mPr*) given the data. If one model has a very large probability and all other models have probabilities close to zero, there is strong evidence for this single model (i.e., a specific combination of predictors). To compare models, we used the package AICcmodavg [89]. We additionally report the evidence ratio (E_0_) calculated as the ratio of the probability of the chosen model to the probability of the null model. E_0_ therefore indicates how many times the chosen model was more probable than the null model.

##### Head and Ear Movements and Postures

The potential fixed effects were the phase (factor with three levels: pre-stimulus, stimulus, and post-stimulus), valence (factor with two levels: presumed negative, dog, and presumed positive, sheep), intensity (factor with two levels: low and high), distance (continuous: foreground, middle ground, and background), amount of habituation (factor with three levels: 0, 5, and 9 times), and side of the sheep video projection (factor with two levels: right and left). We treated distance as continuous but included the square of the distance in some models in order to differentiate whether the relationship is linear or not. Additionally, the proportion of available data for each phase was used as a weight in the evaluation such that more complete phases were weighted more strongly. The random effects included the trial, nested in block, nested in (daily) session and again nested in sheep identity. The outcome variable “proportion of both ears passive” was dichotomized as being 100% or less, because the model estimates did not fit the box plot of the raw data well when plotted. We saw that sheep had either passive ears all the time or almost never. The outcome variable “proportion of both ears backwards” occurred too rarely to allow statistical analysis.

To select the best-fitting model(s), we defined a fixed set of likely models. The maximum model included all the fixed effects (listed above) and some interactions among these fixed effects. Because we expected that the changes from the pre-stimulus phase to the stimulus phase and the post-stimulus phase would be modified by the specific stimuli, the listed fixed effects were included in an interaction with phase in all the models (Table 1). We hypothesized that the type of dog and sheep stimuli presented would change the temporal course of the reactions with stronger reactions when the animal(s) in the video were closer and representing a high intensity stimulus. Moreover, we expected that the specific characteristics of the dog and sheep videos (i.e., distance of the individuals in the videos and intensity) would modulate each other’s effect. We also assumed that the amount of habituation would alter the temporal course of the reactions because unhabituated sheep would be more subject to novelty and might therefore react more strongly (Model e, Table 1). For lateralized responses, i.e., the proportion of time that sheep looked towards the right side, the proportion of time with left-asymmetric ears, and the side where dog and sheep videos were projected, might be important too (Model d, Table 1).

We also included a model that included most of the interactions between dog and sheep stimuli but would allow for a non-monotonous effect of the distance (model g, Table 1). Then, we included some simplified models (Models e, f, and h–l, Table 1). We complemented the model set with a model that included an effect of the *Phase* only assuming that animals react to the stimuli in general (model m), and with a model including the intercept only, that is, a null model without fixed effects (model n).

##### Frontal Cortical Activity

For [O_2_Hb] and [HHb], lmer did not allow a direct modelling of temporal correlations within a stimulus [12,22]. Therefore, we accounted for a high one-step temporal auto-correlation in our 1 Hz recordings by averaging our fNIRS data across 3 s, resulting in two, ten, and two values for the pre-stimulus (6 s), stimulus (30 s), and post-stimulus (6 s) phases, respectively. The emerging fNIRS dataset was potentially composed of 19 sheep × 72 types of stimuli × 8 light paths × 14 values throughout each stimulus (153,216 observations). We realized 150,486 observations that could be analyzed (98.22%). This reduction was due to the exclusion of some stimuli and paths because of movement artefacts (1.34%) or recording failure (0.44%). The potential fixed effects were the same as for the head and ear movements and positions: the time course (continuous variable as a natural spline), valence (factor with two levels: presumed negative, dog, and presumed positive, sheep), intensity (factor with two levels: low and high), distance (continuous, as above, factor with three levels: foreground, middle ground, and background), amount of habituation (factor with two levels: 5 and 9), side of the sheep video projection (factor with two levels: right and left). We used 5 degrees of freedom for the spline modelling time (see Reference [47]). These degrees of freedom allowed a smooth curve changing from a baseline and back with some additional freedom for further, more detailed temporal changes. Given the sensor configuration, we could additionally include head laterality (factor with two levels: left and right hemisphere), longitudinal position (factor with two levels: cranial and caudal location) and measurement depth (factor with two levels: deep and superficial measurement) as fixed effects. The random effects included the single paths (all eight possible combinations of right-left, cranial-caudal, and deep-superficial), nested in trial, nested in block, nested in (daily) session, and again nested in sheep identity. To select the best-fitting model(s), we again used the same defined fixed set of likely models (models d–n). We included an additional more complex model that reflected the main hypothesis we had for the additional fixed effects possible for the fNIRS data (model a). We expected that the cranial-caudal location would change the course of reactions because if we expect to measure frontal cortical reactions, these might be weaker farther to the back compared with the more frontal brain region. We also expected that the deep-superficial path would change the temporal course of reactions because the deep path penetrated more deeply into the tissue and would be more directly influenced by activity changes in the brain. We assumed that the right-left path would vary the temporal course of the reactions because of possible asymmetric processing (model a). In addition, we looked at some of the likely interactions in greater isolation: with a model that included the specific characteristics of the dog and sheep videos (i.e., distance of the individuals in the videos and intensity), the amount of habituation, and the right-left path in interaction with the side where the negative and positive video stimuli were projected throughout the time course (model b), and with a model that included only the right-left path in interaction with the side where the negative and positive video stimuli were projected throughout the time course (model c).

## 3. Results

### 3.1. Approach–Avoidance

In the approach–avoidance paradigm, intra-observer agreement for the behavioral reactions of the sheep was 100%, except for the duration of attention shown towards the presumed negative and positive screens, where agreement was 0.98 and 0.98, respectively.

**Figure 3:**
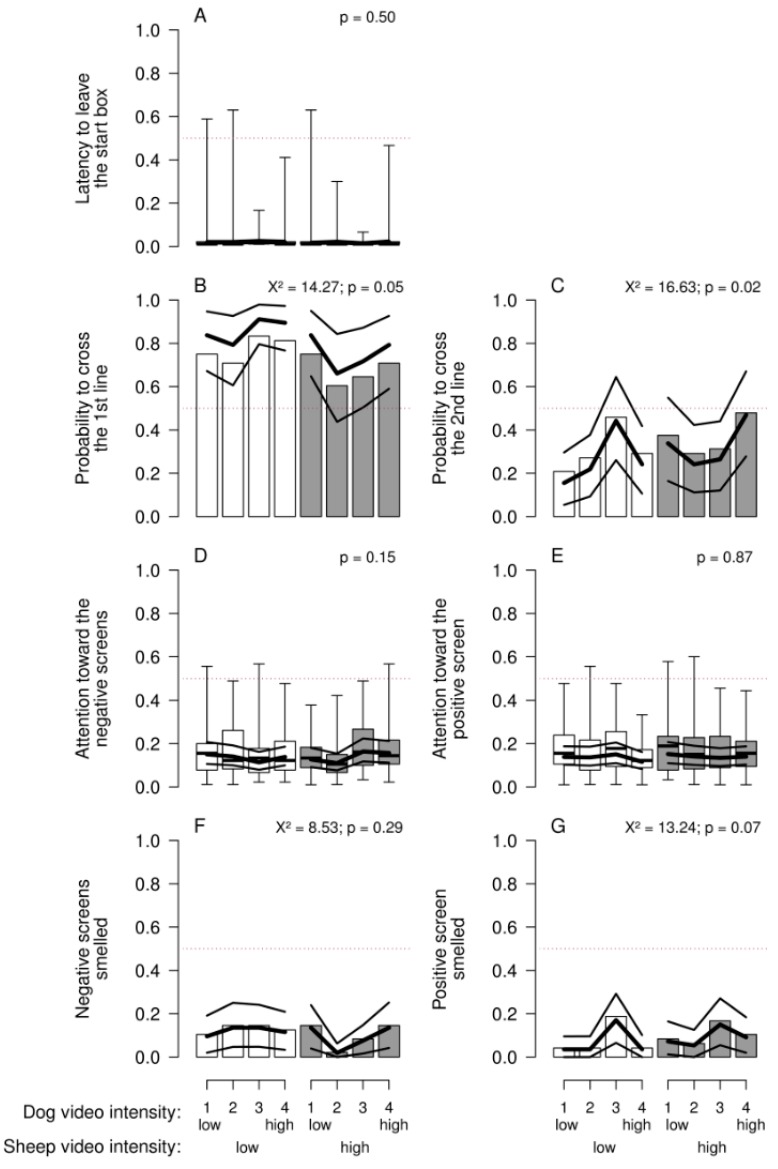
Sheep’s reactions in the approach–avoidance paradigm according to the presumed intensity of the sheep (low: one sheep moving; high: several sheep moving) and dog videos (1 (low): one dog lying; 2: one dog sitting; 3: one dog moving; 4 (high): two dogs moving): (**A**) Latency to leave the start box (proportion of the trial duration); (**B**) probability that the first line was crossed; (**C**) probability that the second line was crossed; (**D**) proportion of trial duration with sheep’s attention towards the negative screens; (**E**) proportion of trial duration with sheep’s attention towards the positive screen; (**F**) probability that the negative screens were smelled; and (**G**) probability that the positive screen was smelled. Boxplots indicate data range as well as median, and lower and upper quartiles. Thick black lines are the model estimates (main effects plus interaction), and thin black lines are the 95% confidence intervals.

With this paradigm, there was no clear evidence that behavioral reactions of the sheep varied consistently according to the presumed valence of the stimulus (dog vs. sheep) or its presumed intensity (model estimates in Figure 3). We found no overall consistent pattern, either, that would suggest a modification of our presumptions. Some *p*-values approached values that would usually be considered “significant” (Figure 3). Given that these low values occurred with the dichotomized outcome variables for which the random effect was simplified, and the models therefore slightly anti-conservative, they should be viewed with caution.

### 3.2. Operant Conditioning

In the habituation phase (week 1), there was no evidence that the number of target touches varied according to the video valence or intensity (Table 2a). No pattern was visible in the raw data nor the model estimates either (not shown).

**Figure 4:**
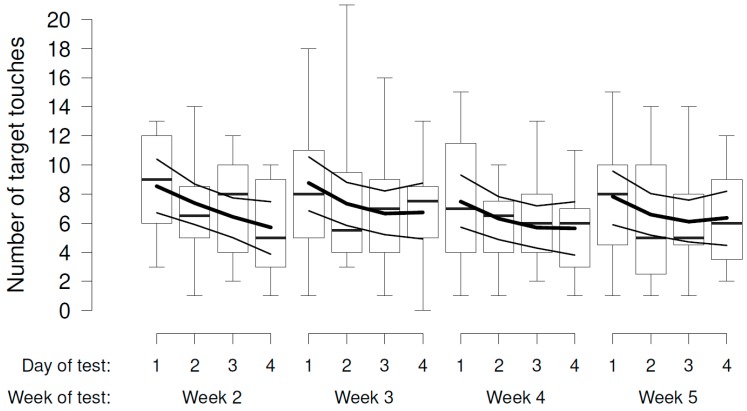
Sheep’s operant responses measured as the number of target touches within the 3 min of each trial during the days of weeks 2–5 towards video stimuli differing in presumed valence and intensity. Boxplots indicate data range as well as median, and lower and upper quartiles. Thick black lines are the model estimates, and thin black lines the 95% confidence intervals.

In the experimental phase (weeks 2 to 5), there was some evidence that the number of target touches decreased across the days of test within the week (Figure 4), but no evidence that it varied according to the presumed valence or intensity of the videos (Figure 4, Table 2a).

**Figure 5:**
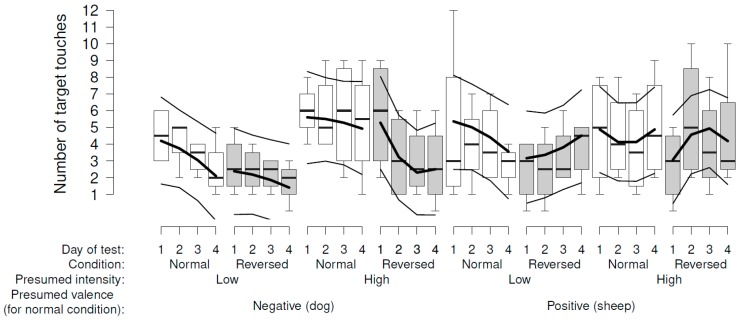
Sheep’s operant responses measured as the number of target touches within the 3 min of each trial during the days of weeks 5 (normal: white boxplots) and 6 (reversed: grey boxplots) towards video stimuli differing in presumed valence and intensity. Boxplots indicate data range as well as median, and lower and upper quartiles. Thick black lines are the model estimates, and thin black lines the 95% confidence intervals.

In the reversal phase, we decided to analyze dog and sheep videos separately because the pattern looked very different, though the interaction between video valence and the effect of target touch (normal vs. reversed) did not reach a very low *p*-value (0.06). With the dog videos, there was evidence that the sheep touched the target less over the days of test, and also when the touch switched on the dog video (reversed) compared with switching off the dog video (Table 2b, Figure 5).

The raw data implied that this effect was mainly driven by a marked reduction of touching the target when it had the effect of switching on the more intensive dog videos, though this interaction was not clearly supported statistically (*p* = 0.14, Table 2b, Figure 5). With the sheep videos, there was no evidence that the sheep’s responses varied consistently according to the day of test or condition of the sessions (Table 2b, Figure 5).

### 3.3. Attention Test

**Figure 6:**
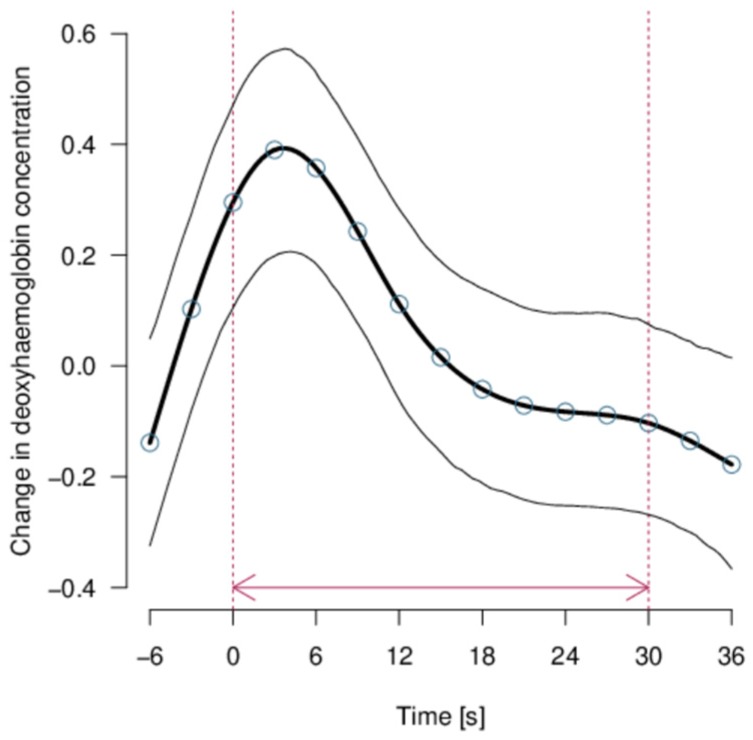
Deoxyhaemoglobin concentration changes on the transformed scale during the video stimuli projection. The stimulus phase is marked by the red arrow between the vertical lines. The thick black line is the model estimate (with the blue circles representing the observed time-values), and thin black lines are the 95% confidence intervals.

No effect of the predictor variables was apparent for [O_2_Hb] given that the null model was the most probable (*mPr* = 1). Strong evidence was found that [HHb] showed a systematic pattern throughout the stimuli (model including the time course only: *mPr* = 1; E_0_ > 20,000), that is, [HHb] showed a peak at the start of presenting the stimuli (Figure 6). However, no consistent modifications of this pattern were observed according to the type of stimulus shown (varying in presumed valence, intensity, and distance), the amount of habituation, the light paths, or the side of projection of the videos.

**Figure 7:**
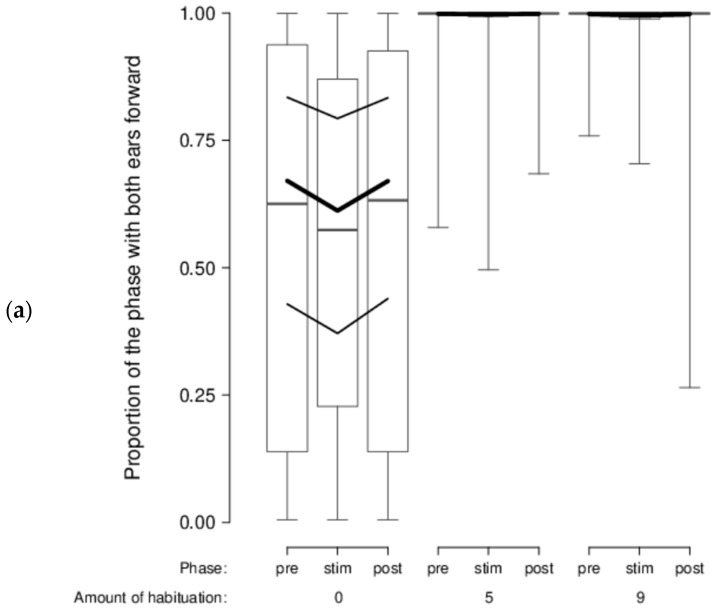

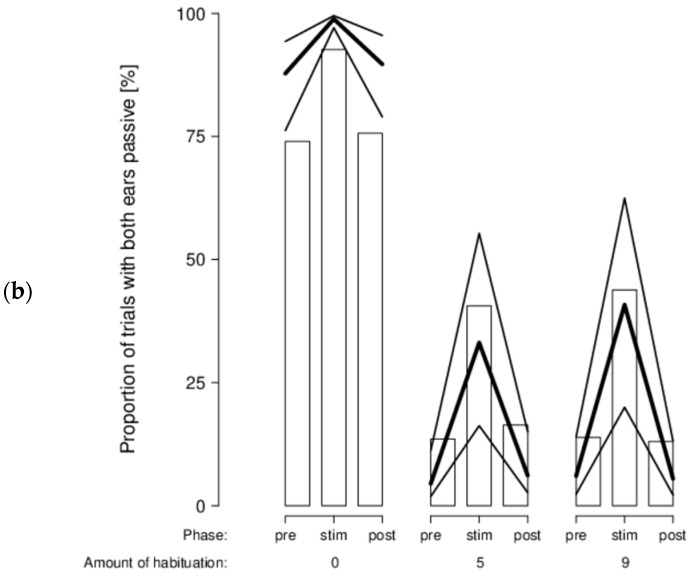
Sheep’s ear positions according to the phase (pre-stimulus, stimulus, post-stimulus) and amount of habituation prior testing (0, 5, 9 times): (**a**) proportion of the phase with both ears forward; (**b**) proportion of trials with both ears passive throughout the phases. Boxplots indicate data range as well as median, and lower and upper quartiles. Thick black lines are the model estimates (including phase, amount of habituation and interaction), and thin black lines are the 95% confidence intervals.

There was no indication that sheep’s head and ear movements and positions varied according to the type of stimuli shown, either. However, we found a clear phase effect (stimulus vs. pre-stimulus and post-stimulus), in that the best-fitting model for all our behavioral variables was the model with only the main effect phase. During the stimulus phase, sheep moved their head more (*mPr* = 0.987; E_0_ > 19,740), switched their head more from one side to the other (*mPr* = 1; E_0_ > 20,000), moved their ears more (*mPr* = 1; E_0_ > 20,000), had a lower proportion of time with their head on the right side (*mPr* = 0.994; E_0_ = 177), a lower proportion of time with ears forward (*mPr* = 0.982; E_0_ > 19,640), a higher proportion of time with passive ears (*mPr* = 0.9998; E_0_ > 19,996), and a lower proportion of time with left-asymmetrical ears (*mPr* = 1; E_0_ > 20,000). In addition, a certain effect of the amount of habituation was visible in the data even if it was much less supported statistically: habituated sheep had both ears forward almost all of the time (second most likely model, model phase*habituation: *mPr* = 0.02; E_0_ > 400; Figure 7a) and a low proportion of time with both ears passive in comparison with unhabituated sheep (second most likely model, model phase*habituation: *mPr* = 0.0002; E_0_ > 5; Figure 7b).

## 4. Discussion

In the present study we wanted to investigate whether sheep would assess video stimuli of dogs and sheep as negative and positive, respectively. We will discuss the findings of the single experiments first and then examine why the type of visual stimuli may not be the best suited to assess stimulus valence in sheep.

The approach–avoidance paradigm did not support the assumption that sheep videos would be more positive than dog videos. We had expected that sheep would be less likely to cross the experimental arena with increasing dog intensity and would pay more attention to the dog videos when these were of higher intensity. However, we did not find consistent effects of the video intensities on the sheep’s reactions. It is commonly assumed that dogs are species that would normally represent some form of threat for sheep [28,46,58,69,70]. Previous studies found that in the presence of a real dog, sheep stayed further away from a group of sheep restrained behind the dog and explored the test arena very little in an approach–avoidance paradigm [18], and sheep are highly vigilant [18,28,46]. Sheep did seem to react differently to dog intensity 3 (i.e., one dog moving), but it is unclear why they did so in the observed way. Our test might not have worked as well as expected because the tested sheep could have been equally interested in the sheep and dog video stimuli and thus approached both. This is not surprising for the conspecifics given that sheep are social animals. Even if the dog is perceived as a potential threat, it may make sense to pay attention to its presence. Alternatively, sheep might have quickly learnt that the animals in the videos were unresponsive, i.e., not interacting socially nor presenting a real threat.

The results from the reversal phase in our operant conditioning approach were consistent with our hypothesis that sheep were more prone to avoid dog videos, especially when these were of high intensity. With this approach, we would also have expected that sheep would be more motivated to watch the more intensive sheep videos and less interested to see the high intensity dog videos. Yet, we found little evidence for these aspects. We may not have found a stronger effect in the reversal phase of this experiment because only ¼ of the subjects (i.e., four sheep) were tested in each specific condition. In this view, a future experiment should replicate this last part (i.e., the reversal phase) with a larger sample size. All the same, we found a strong mean decrease in sheep responses to dog videos in week 6, when they did switch on the dog videos instead of switching them off.

In the attention test, we found that sheep clearly reacted to the presentation of the stimuli, both in their behavior and in their frontal brain haemodynamic, indicating that they did pay attention to these stimuli. [HHb] was increased at the onset of the videos indicating a frontal cortical brain deactivation. This confirms previous studies that found a frontal neural inhibition caused by visual stimuli [48,90]. We expected that the sheep’s behavioral or physiological reactions would vary according to the stimuli valence, intensity, and distance, however, we were not able to support such a notion. This conflicts with the study of Vögeli, Wolf, Wechsler, and Gygax [48] where sheep attentiveness decreased from presumed negative video sequences (agonistic interactions in conspecifics) to presumed positive ones (ruminating and co-feeding sheep). We may not have found this same pattern because our stimuli were too weak. This is unlikely, however, as a dog seems to be much more threatening than sheep involved in an agonistic interaction. During the stimulus phase (vs pre- and post-stimulus), our sheep showed more ear movements, both ears forward often, and few passive ears, especially when habituated. In sheep, more ear posture changes [13,14,47,49,52] and forward ears [13,14,47,54,91] have often been associated with negative valence. This would indicate that habituated sheep found the video stimuli more aversive than unhabituated sheep, which seems counterintuitive. However, Briefer, Tettamanti, and McElligott [11] associated ears pointed forward and few passive ears in goats with high arousal rather than with a specific valence. We observed ears forward for a large proportion but also passive ears for a considerable amount of time. The according arousal state of our sheep therefore remains unclear. Moreover, our sheep also had a lower proportion of time with their head on the right side during the stimulus phase that could reflect a small shift in laterality; sheep were looking slightly more with the left eye. This finding is consistent with left eye preferences (i.e., right hemisphere bias) found in cows [92,93], goats [94], and horses [95,96] responding to threats or novel stimuli. Tracking sheep’s head and ear movements and positions might not be ideal to measure visual attention because of the wide visual field of sheep. Therefore, using an eye tracking system might be an improvement [97,98,99,100,101]. However, it is unclear whether such a system could be calibrated to be used in species with a large visual field. In this third experiment, we used a complex design with stimuli varying in valence, individual distances, and rate of movements, resulting in a high number of combinations as trials that may have led to habituation, such that the stimuli were no longer of interest to the sheep.

A major issue with this study was the type of stimuli we used, that is, video images. One explanation for the results of the three experiments could be that our sheep might have had difficulty in distinguishing the visual social stimuli. It may be that sheep did not distinguish between dogs and sheep in the way we expected, but treated all quadrupeds as potential threats. However, previous studies have shown that sheep are able to discriminate between sheep and dogs’ face images [58,59]. Yet, it has not been investigated independently how well sheep perceive video images like the ones used. However, Vögeli, Wolf, Wechsler, and Gygax [48] and Bellegarde [91] assessed sheep reactions to videos of conspecifics in situations of varying valence (e.g., agonistic interactions, rumination, from front and side views) and found that sheep reacted to the situations differently. Also, video stimuli can be perceived as deficient relative to live conspecifics in (absence of) odors, colors, illumination, background and contrast, and sounds and vibrations [60,102,103,104]. Studies investigating sheep’s attention in response to a real dog (sitting quietly and visible through a window) found clear behavioral responses [28,46]. However, we decided to use videos in order to control the presentation, repeat identical stimuli to the different subjects, and reproduce the features of complex real stimuli [103,104]. We used silent video images because we were interested in the role of visual stimuli in the sheep’s attention shift. As explain by D′Eath [104], it is important to ensure that the visual component causes the observed reaction and not the sound alone. If sound plays a role in communication in animals [60,105], previous studies found that the auditory components in video images was not essential ([56,106], and for a review see Reference [104]). However, there are also limitations to using video images of stimuli [103,104]: (1) the stimuli are two-dimensional images with a lack of depth; (2) the stimuli do not interact with the subject, that is, the animals in the videos do not respond nor present a real threat; and (3) depending on the visual capabilities of a species, a video may be perceived as a quick succession of single pictures instead of the illusion of a continuous moving image. Indeed, many animals have vision that is very different from that of humans and an image that looks excellent to us may be unrecognizable to them [107]. The critical fusion frequency of a sheep has been established above 80 Hz [67], therefore clearly higher than that of humans (36–50 Hz in humans [107,108]). In our study, videos were recorded and then projected using the Phase Alternating Light (PAL) standards (50 Hz, 25 fps), which implies that the sheep vision refresh rate was higher than the number of successive pictures available per second. While a high critical fusion frequency may lead to a perception of flicker, the illusion of smooth continuous moving images does not depend on it, but on the degree of movement and displacement between successive images (for more details see [107]). Nevertheless, Fleishman and Endler [107] argue that flicker may be aversive or distracting for tested animals. In our study, the temporal resolution should have been sufficient, because the displacement of the animal(s) in successive frames was relatively small. This is supported by our operant conditioning experiment that provided some evidence for sheep discriminating between videos of sheep and dogs. Nevertheless, we would recommend using video with higher frame rates for further experiments in sheep. As an alternative to higher frame rates, another sensory channel could be used, such as acoustic stimuli (e.g., sheep bleating and dog barking). In addition, sheep may show more clearly where their attention is focused with acoustic than silent visual stimuli because their hearing may be more directed towards stimuli than their vision, as seen in the ear movements.

## 5. Conclusions

The current study shows that sheep react to the presentation of video stimuli, indicating that they pay attention to these. However, with all our approaches, it was surprisingly difficult to support the notion that sheep’s attention to the video stimuli used varied according to their valence and intensity. It was difficult to measure sheep’s differential attention and only the operant conditioning approach indicated that our sheep were more willing to avoid the dog videos, especially when the dog in the video was moving. Overall, we found that standard video images may not be ideal to represent valence characteristics of stimuli to sheep. For further studies, we recommend using another perceptual channel to assess the valence of stimuli in sheep.

## Figures and Tables

**Table 1:** List of the models used for the statistical analyses of the head and ear position and movements (d–n) and concentration of oxyhaemoglobin ([O_2_Hb])/concentration of deoxyhaemoglobin ([HHb]) (a–n). *Phase*: time course (functional near-infrared spectroscopy (fNIRS) data) or phase (behavioral data), *di*: dog video intensity, *dd*: dog video distance, *si*: sheep video intensity, *sd*: sheep video distance, .*sqa*: quadratic term, *SrS*: side of the sheep video, *nT*: amount of habituation, *rile*: right-left path, *prodis*: cranial-caudal path, *sholo*: deep-superficial path, * include the main effects and all the possible interactions among the predictors separated by ‘*’ (as with the formula notation in R).

Models Used	
*a*	Di *dd *si *sd *Phase + prodis *Phase + sholo *Phase + rile *SrS *Phase + nT *Phase
*b*	Dd *di *sd *si *Phase + rile *SrS *Phase + nT *Phase
*c*	Rile *SrS *Phase
*d*	Dd *di *sd *si *Phase + SrS *Phase + nT *Phase
*e*	Dd *di *sd *si *Phase + nT *Phase
*f*	Di *dd *Phase + si *sd *Phase + dd *sd *Phase + di *si *Phase
*g*	Di *dd.sqa *Phase + si *sd.sqa *Phase + dd.sqa *sd.sqa *Phase + di *dd *Phase + si *sd *Phase + dd *sd *Phase + di *si *Phase
*h*	Dd *sd *Phase
*i*	Si *di *Phase
*j*	Si *sd *Phase
*k*	Dd *di *Phase
*l*	nT *Phase
*m*	*Phase*
*n*	*1 (null model)*

**Table 2:** Operant conditioning: statistical information (Χ^2^; *p*) of the effects on the number of target touches: (**a**) Results for the habituation phase, experimental phase, and reversal phase; (**b**) Detailed statistical results of the effects of target touches for the dog and sheep videos during the reversal phase.

Phase or Video Type		Global	Effect of Target Touch	Video Valence	Video Intensity	Day of Test	Interactions
(a)	**Habituation phase**	13.69; 0.44	NA	-	-	-	-
	**Experimental phase**	19.11; 0.07	NA	1.44; 0.23	0.07; 0.79	14.65; 0.003	All interactions ≤0.53; ≥0.78
	**Reversal phase**	37.61; 0.08	5.83; 0.06	−0.40; 1	0.99; 0.41	3.57; 0.21	Effect * Valence 4.28; 0.06 Day of test * Valence 5.52; 0.08 All other interactions ≤4.27; ≥0.11
(b)	**Dog videos**	43.92; 0.001	17.76; 0.002	NA	1.02; 0.42	14.39; 0.003	All interactions ≤1.53; ≥0.14
	**Sheep videos**	7.28; 0.85	-	NA	-	-	-

* include the main effects and all the possible interactions among the predictors separated by ‘*’ (as with the formula notation in R).

## Supplementary Materials

The following are available online at http://www.mdpi.com/2076-2615/8/7/121/s1. File S1: Approach-avoidance paradigm, operant conditioning and attention test (fNIRS and movements’ tracking)’s raw data and outcome variables, fixed effects, and random effects’ list definitions.
